# Relationship Functioning and Gut Microbiota Composition among Older Adult Couples

**DOI:** 10.3390/ijerph20085435

**Published:** 2023-04-07

**Authors:** Qiwen Cheng, Rosa Krajmalnik-Brown, John K. DiBaise, Juan Maldonado, M. Aaron Guest, Michael Todd, Shelby L. Langer

**Affiliations:** 1 Biodesign Center for Health through Microbiomes, Arizona State University, Tempe, AZ 85281, USA; qcheng16@asu.edu (Q.C.); dr.rosy@asu.edu (R.K.-B.); 2 School of Sustainable Engineering and the Built Environment, Arizona State University, Tempe, AZ 85281, USA; 3 Division of Gastroenterology and Hepatology, Department of Internal Medicine, Mayo Clinic College of Medicine and Science, Scottsdale, AZ 85259, USA; dibaise.john@mayo.edu; 4 Knowledge Enterprise Genomics Core, Arizona State University, Tempe, AZ 85281, USA; juanmaldonado.ortiz@gmail.com; 5 Center for Innovation in Healthy and Resilient Aging, Edson College of Nursing and Health Innovation, Arizona State University, Phoenix, AZ 85004, USA; aaron.guest@asu.edu; 6 Edson College of Nursing and Health Innovation, Arizona State University, Phoenix, AZ 85004, USA; 7 Center for Health Promotion and Disease Prevention, Edson College of Nursing and Health Innovation, Arizona State University, Phoenix, AZ 85004, USA

**Keywords:** couples, relationship satisfaction, intimacy, gut microbiome, gut microbial diversity

## Abstract

An emerging area of research extends work on couple functioning and physical health to gut health, a critical marker of general health and known to diminish with age. As a foray into this area, we conducted a pilot study to (1) determine the feasibility of remote data collection, including a fecal sample, from older adult couples, (2) examine within-couple concordance in gut microbiota composition, and (3) examine associations between relationship functioning and gut microbiota composition. Couples (N = 30) were recruited from the community. The participants’ demographic characteristics were as follows: M (SD) age = 66.6 (4.8), 53% female, 92% White, and 2% Hispanic. Two of the couples were same-sex. All 60 participants completed self-report measures and supplied a fecal sample for microbiome analysis. Microbial DNA was extracted from the samples, and the 16S rRNA gene V4 region was amplified and sequenced. The results indicated that individuals shared more similar gut microbial composition with their partners than with others in the sample, *p* < 0.0001. In addition, individuals with better relationship quality (greater relationship satisfaction and intimacy and less avoidant communication) had greater microbial diversity, *p* < 0.05, a sign of healthier gut microbiota. Further research with a larger and more diverse sample is warranted to elucidate mechanisms.

## 1. Introduction

An extensive literature on health and social relationships suggests that social ties are salutary [1]. Social isolation, by contrast, is a risk factor for poor physical health, ranging from greater susceptibility to the common cold [2] to greater odds of mortality [3,4]. Effect sizes from large-scale prospective studies linking social isolation to morbidity and mortality are comparable, in fact, to those for well-established risk factors such as smoking [4].

We focus here on a specific type of social tie, intimate cohabiting relationships. Marriage in particular has been found to be health-protective across a large number of samples, clinical endpoints, and biomarkers [5]. For example, in the cancer arena, married versus unmarried patients are less likely to present with metastatic disease and to die of the disease [6]. Being married per se, however, is not necessarily protective. The health benefits of marriage are stronger for males than females [6,7]. Relationship quality also matters [8]. Greater marital satisfaction has been associated with better self-reported health, better sleep, fewer physical symptoms, fewer physician visits, less disability, and lower mortality [7,9]. It has also been associated with more adaptive health behaviors such as better compliance with medical regimens [7].

A related literature provides strong support for the notion that members of a couple are similar or concordant with regard to health status and health behaviors. For example, within-couple concordance has been observed for weight [10], BMI [10,11], waist/hip ratio [10], and blood pressure [10]. It has also been observed for perceived health [12], depressive symptoms [12,13], and health behaviors ranging from smoking [10,11] to dietary intake [11] and exercise frequency [11]. One explanation for these associations is assortative mating, the tendency to select a mate with like characteristics including sociodemographic factors, attitudes, and behavioral patterns [14,15]. Couples also share spaces/environments and one partner (often the female in different-sex relationships) is likely to exert influence on the other, termed social control [16]. For example, one partner might attempt to regulate the other’s dietary intake.

Within-couple concordance extends to a biomarker receiving increasing attention in medicine, that of the gut microbiome [17]. Gut microbiota consist of thousands of microorganisms including bacteria, archaea, and fungi [18], and play a key role in digestion and immune functioning [17]. The composition of gut microbiota matters. Diversity is adaptive and lack of diversity, maladaptive. Lower bacterial diversity has been linked to risks for obesity, dyslipidemia, insulin resistance, inflammation, and gastrointestinal disorders such as irritable bowel syndrome and inflammatory bowel disease [17,19,20]. Gut microbial composition is influenced by social and environmental factors. It is more similar among cohabitants than non-cohabitants [21]. Evidence to date primarily comes from research with animals. Studies with non-human primates reveal similar gut microbial communities among those in closer proximity and with more contact time [22]. In addition, within social groups, close grooming partners have more similar core microbiota than those who rarely groom one other [23,24,25]. Research with humans similarly indicates that cohabiting family members share microbiota with one another [26,27], especially couples [26]. As with other health states and health behaviors, concordance may be explained by similar environments [28] and diets, but unlike blood pressure or physical activity, concordance of gut microbiota may be explained by microbial sharing as a function of proximity [28], common surfaces [28], and close contact [25].

Dill-McFarland and colleagues examined associations between social relationships and gut microbiota [29] using data from the Wisconsin Longitudinal Study, a prospective cohort of Wisconsin high school graduates (N = 10,317). Participants were first assessed in 1957 and repeatedly thereafter. Their significant others were also assessed, namely siblings (in 1977, 1994, 2005, and 2011) and spouses (in 2004 or 2006). The Dill-McFarland et al. sample comprised 408 individuals: 179 graduates, 134 siblings of graduates, 63 spouses of graduates, and 32 spouses of siblings. Major findings related to the present study were as follows: (1) The gut microbiota of cohabiting spouse/partnered pairs were more similar in composition as compared to those of sibling pairs and unrelated participants. (2) Among spouses, the length of cohabitation was positively correlated with similarity. (3) The gut microbial composition of cohabiting spouses/partners was more diverse and richer than that of persons living alone. (4) Within the cohabiting married group, gut microbial composition was especially diverse among those reporting to be in closer relationships, with closeness assessed by a single item: “How close are you and your current spouse?” The gut microbial diversity of participants reporting to be in very close relationships was greater than that of persons living alone, but that of participants in somewhat close relationships was not. As suggested by the authors, proximity alone may not explain the greater diversity among those in closer relationships [29]. Relationship intimacy likely plays a role.

While innovative, informative, and leveraging an existing cohort, the Dill-McFarland et al. study [29] used a single (unvalidated) item to assess relationship closeness. The investigators also did not assess other dimensions of relationship functioning such as relationship satisfaction, intimacy, or how couples communicate, which have been found to predict psychological and relational well-being [30] and even survival [9]. In addition, the study was based on a non-diverse sample from the Midwest including no same-sex couples, and relationship partners were recruited via available cohort participants and their siblings, rather than a priori sampling and recruiting of intact couples. Moreover, to our understanding, subgroups of participants were assessed at different points in time between 1957 and 2015.

We sought to advance this emerging line of research in multiple ways. First, we pilot-tested the feasibility of collecting fecal samples from older adult partners using remote methods (necessitated in part by study timing, during the early phases of the SARS-CoV-2 pandemic). Second, we examined the concordance of microbial diversity and composition in a national sample of older adult spouses. Third, we examined associations between gold standard measures of relationship functioning and gut microbial composition. To our knowledge, no study has examined these associations but there is a rationale for doing so, especially among older adult couples. As noted above, marital satisfaction has been linked to better health. In contrast, marital conflict has been associated with cardiovascular, immune, and endocrine dysregulation [7], and perceived spousal criticism is a risk factor for mortality [31]. The impacts of marital discord, moreover, are greater for older versus younger couples [32]. Older adults are more reactive to disagreements with their partner and may have more limited social contacts, thus placing greater emotional weight on their spousal relationship [32]. Interactions characterized by avoidance can also be maladaptive. Withholding or lack of disclosure has been associated with lower relationship satisfaction [33] and adverse medical outcomes in prospective studies [34], and older adults may be more likely to avoid conflict [32]. Lastly, the aging population is at risk for age-related decrements in gut microbial composition associated with inflammation and immune decline [32,35].

## 2. Materials and Methods

The study was cross-sectional in design. All procedures were approved by the Institutional Review Board of Arizona State University (study #00012144), and all methods were performed in accordance with the ethical principles for medical research involving human participants outlined in the Declaration of Helsinki. 

Restrictions related to the SARS-CoV-2 pandemic necessitated shifting from originally planned in-person participant recruitment and fecal sample collection to remote processes. Participants were recruited through social media and by approaching individuals in a larger study of older adults who had agreed to be contacted for future research. The inclusion criteria were age 60+, marriage or cohabiting partnership, and English speaking/understanding. The exclusion criteria were a gastrointestinal disorder, receiving enteral nutrition (i.e., “tube feeding”), use of antibiotics in the past month, cancer treatment in the past 6 months, a positive COVID-19 diagnosis in the past two months, and currently residing outside the US.

The consent process was initiated by phone, with a research coordinator describing the study’s purpose, procedures, risks, and benefits. Individuals expressing willingness to participate were then sent a unique email link to access the full consent form through Research Electronic Data Capture (REDCap), with an option to sign digitally and download a pdf version of the signed form for their records. Those completing this signing process proceeded automatically to the one-time survey, also administered via REDCap. The self-report measures are described in turn below.

### 2.1. Self-Report Measures

*Relationship quality*. To assess relationship quality, we administered the 10-item relationship satisfaction subscale of the Dyadic Adjustment Scale [36]. Example items include, “How often do you think that things between you and your partner are going well?” and “How often do you discuss or have you considered divorce, separation, or termination of your relationship?” (reverse-scored). Subscale scores can range from 0 to 50 with higher values indicative of greater satisfaction. Cronbach’s alpha for this sample (N = 60) was 0.94, indicating high internal consistency. To assess intimacy, we administered the 17-item Miller Social Intimacy Scale [37]. Six items assess the frequency of relevant behaviors, e.g., “How often do you show your partner affection?”, and 11 items assess intensity, e.g., “How close do you feel to your partner most of the time?”. Mean scores range from 1 to 10 with higher values indicative of greater intimacy. Cronbach’s alpha for this sample was 0.88, indicating high internal consistency.

*Couple communication*. We assessed open sharing of thoughts and feelings, and holding back from such sharing, with a modified version of the Emotional Disclosure Scale [38]. This measure asks respondents to rate the extent to which they talked to their spouse about 7 different concerns: concerns about their physical health; concerns about their emotional well-being; their own negative feelings, such as fear, worry, or sadness; concerns about their relationship with their spouse; concerns about their relationship with others (family members, friends); job-related concerns; and financial concerns. We added an 8th item, concerns about the coronavirus or COVID-19. A parallel set of items asks respondents to rate the extent to which they held back from talking to their spouse about the same concerns; these constitute the holding back subscale. All ratings are made on a 1–5 (not at all to a lot) scale and averaged. Higher values indicate greater levels of the construct. Cronbach’s alpha values were 0.85 and 0.88 for disclosure and holding back, respectively.

We also administered the Communication Patterns Questionnaire [39] which prompts respondents to consider how they and their partner behave (a) when a problem in their relationship arises, (b) during discussion of a relationship problem, and (c) after discussion of a relationship problem. Nine items such as “both my partner and I try to discuss the problem,” “both my partner and I suggest possible solutions and compromises,” and “both my partner and I feel understood by the other” are averaged to assess constructive communication. Ratings are made on a 1–9 (very unlikely to very likely) scale. Cronbach’s alpha was 0.83.

*Sexual satisfaction*. A single item was used to assess sexual satisfaction: Please rate the extent to which sexual activity has been satisfying for you in the past month, from 0 to 10, with 0 being not at all satisfied and 10 extremely satisfied.

*Dietary intake*. Dietary intake information was gathered using the NCI Dietary Screener Questionnaire (National Health and Nutrition Examination Survey 2009-10) [40] and scored following the guidelines described at https://epi.grants.cancer.gov/nhanes/dietscreen/scoring/current/ (accessed on 24 July 2021). We focused on the daily consumption of fiber, calcium, and added sugars because these are known to affect the gut microbiota [41,42,43,44,45].

*Medications and chronic conditions*. Participants were asked to indicate whether they currently take certain medications that can affect gut microbiota, including the following acid-reducing medications: Prilosec, Prevacid, Protonix, Aciphex, Nexium, Dexilant, Zantac, Pepcid, or Tagamet. They were also asked to indicate whether they currently take a prebiotic such as fructo- and galacto-oligosaccharides, or a probiotic such as Lactobacillus, Bifidobacterium, Align, or Culturelle. The prebiotic and probiotic use was grouped as “supplement use” in microbiota analyses, with “0” representing no use at all and “1” use of either or both.

We used the Charlson Comorbidity Index [46] to assess chronic disease conditions. Participants were asked, “As far as you know, do you have any of the following health conditions at the present time?” The list included 10 conditions: asthma, emphysema, or chronic bronchitis; arthritis or rheumatism; cancer (diagnosed in the past 3 years); diabetes; digestive problems such as ulcer, colitis, or gallbladder disease; heart trouble such as angina, congestive heart failure, or coronary artery disease; HIV illness or AIDs; kidney disease; liver problems such as cirrhosis; and stroke. We added an 11th item to assess the presence of COVID-19. Affirmative responses were coded “1” and negative responses “0”. For the microbiota analysis, the disease conditions were further grouped to “0” (no disease) and “1” (at least one disease).

### 2.2. Fecal Sample Collection, Sequencing, and Microbiota Analysis

Fecal samples were collected using the OMR-200 collection kit (DNAgenotek, Inc., Kanata, ON, Canada). This kit was chosen for its ease of use, simplicity, and because samples can be stored at room temperature for up to 60 days, thus negating the need for special storage or shipping. Participants received the kit via United Parcel Service (UPS), including step-by-step collection instructions, a study-provided specimen bag, and a study-provided return envelope. Each participant was asked to provide one fecal sample. Participants were directed to their nearest UPS for return (or provided UPS pick-up if requested); the samples were shipped to Arizona State University and stored in a −80 °C freezer. Prior to DNA extraction, the samples (from the −80 °C freezer) were thawed at room temperature and vortexed vigorously for 10 s. DNA was then extracted from 250 μL of each sample using the MagAttract^®^ PowerSoil Pro^®^ DNA kit (QIAGEN). The V4 region of the 16S rRNA gene was amplified with the primers 515F (5′-GTGCCAGCMGCCGCGGTAA) and 806R (5′-GGACTACHVGGGTWTCTAAT) [47], and sequenced using the Illumina MiSeq platform with a paired-end sequencing of 250 base pair lengths [48].

QIIME 2 (version 2021.2) [49] was used for sequence quality control, feature table construction, and phylogenetic tree generation. The DADA2 plugin [50] was used to filter and merge the forward and reverse reads. Sequences were then mapped to the Silva 138 SSURef NR99 database for microbiota composition analysis. A classifier was trained with the forward and reverse primers used in this study. The relative abundances of microorganisms in each sample were exported at the phylum and genus levels. The relative abundances of individual taxa relative to the abundances of total taxa were visualized using the R ggplot2 package (version 3.3.6) [51] and taxa with low relative abundances (<5%) were grouped as ‘‘others”.

The feature table, taxonomy, and rooted tree files from QIIME2 and metadata were imported into R as a phyloseq object using the qiime2R package (version 0.99.6) [52], and then rarified using the phyloseq package (version 1.40.0) [53] for alpha and beta diversity analyses. The rarefaction depth was 27,105, which was the size of the smallest library of all samples. Alpha diversity measures the number of unique taxa (richness) and how equally abundant the taxa are (evenness) in an individual sample [54]. Beta diversity measures dissimilarity in microbial composition between samples [54]. The alpha diversity metrics, Chao1, Shannon’s diversity, and Faith’s phylogenetic diversity (Faith’s PD), were calculated using the R packages microbiome (version 1.18.0) [55] and picante (version 1.8.2) [56]. The beta diversity matrices, Jaccard dissimilarity and Bray–Curtis dissimilarity, were calculated using the R rbiom package (version 1.0.3) [57].

### 2.3. Analytic Plan

All statistical analyses were conducted in R (version 4.2.0) [58] with the exception of descriptive analyses to characterize the sample with regard to self-report variables; these were conducted using IBM’s Statistical Package for the Social Sciences (version 28). *p*-values of less than 0.05 were considered statistically significant.

Pearson product–moment correlations for associations between alpha diversity metrics and psychosocial measures were computed using the cor function in R. Univariate outliers in alpha diversity were identified with the boxplot.stats function in R with default settings. To compare “within couples” and “non-couples” in beta diversity, the permuted Brunner–Munzel test was conducted using the brunnermunzel package (version 2.0) [59] in R. This distribution-free (nonparametric) analysis of differences between groups is robust to imbalances in group sizes and group variances [60]. To examine associations between beta diversity indices and the psychosocial measures, permutational analyses of variance were conducted with the “adonis2” function (permutations = 9999, other settings as default) in the vegan R package (version 2.6–2) [61]. The UPGMA (unweighted pair group method with arithmetic mean) hierarchical cluster analysis was performed using R’s hclust function.

Variable selection was performed to identify psychosocial characteristics associated with gut microbiota composition, using a forward stepwise method. First, the characteristics measured in this study were pre-processed for this analysis. Ethnicity and race were removed from this step because over 90% of the participants were non-Hispanic White. Gender, income, education, proton pump inhibitor use, supplement use, and chronic disease were coded as categorical variables, and other characteristics as continuous variables, which were scaled to zero mean and unit variance using the decostand function of the vegan package. Multicollinearity was checked using the pairs.panels function of the psych package (version 2.2.5) [62], and all Pearson *r* values were less than 0.7, indicating low collinearity among variables. Next, the Bray–Curtis distance-based redundancy analysis (db-RDA) was conducted using the vegan package’s capscale function, with the variables of interest set as constraints (Table S1). Variation inflation factors calculated using the vegan’s vif.cca function were all less than 5. Variable selection was then performed for the db-RDA ordination using the vegan “ordiR2step” function (R2permutations = 9999, other settings as default). As three non-psychosocial variables (gender, income, supplement use) were selected, which contributed significantly to the microbial composition variation, a second db-RDA was conducted by setting these non-psychosocial variables as “condition” to remove their impact, and psychosocial variables as constraints. A second “ordiR2step” selection was then performed to identify the more predictive psychosocial variables.

To further investigate microorganisms associated with the psychosocial variables identified in the forward selection, we estimated a linear regression using the glm function in the basic stats package. In the rarefied data, core microbiota (relative abundance > 0.1%, prevalence > 50%) in all participants were first identified at the amplicon sequence variant (ASV) level using the R microbiome package (version 1.14.0) [55]. The count of each ASV was modeled with fixed effects of holding back, gender, income, and supplement use (identified in the first forward variable selection step), with the family set as quasipoisson (link = “log”) to allow overdispersion. We did not incorporate the effect of interactions among these variables due to the sample size. This model (i.e., full model) was then compared with a null model without the effect of holding back using R’s anova function (test = “F”), with *p*-values adjusted for multiple comparisons using the Benjamini and Hochberg (BH) method with the R stats package. The model equations are shown below.

Full model: ASV count=holding back+gender+income+supplement use

Null model: ASV count=1+gender+income+supplement use

## 3. Results

### 3.1. Remote Recruitment and Assessment of Older Adult Couples, including Collection of Fecal Samples, Is Feasible

Participants were recruited through multiple sources as noted previously. Of the 41 persons responding with interest across recruitment sources, 32 were contacted for screening. A total of 30 of 31 eligible couples consented. Enrollment and assessment were completed within a three-month period.

Participants (N = 60 individuals, Table 1) ranged in age from 60 to 79. While roughly half of the overall sample identified as female, the majority (83%) of “index” participants (those who responded to recruitment ads or emails and then involved their partner) were female. All couples were married. Two couples were same-sex.

Table 2 lists the psychosocial and behavioral characteristics of the sample. Measures of relationship satisfaction and intimacy indicated that relational well-being was, on average, moderate to high. Forty-three percent of participants reported at least one chronic health condition and approximately one-quarter reported the use of a proton pump inhibitor (which can affect gut microbiota composition and function), though none daily.

### 3.2. Gut Microbiota of Couples Share More Similarity Than Those of Non-Couples

**Figure 1:**
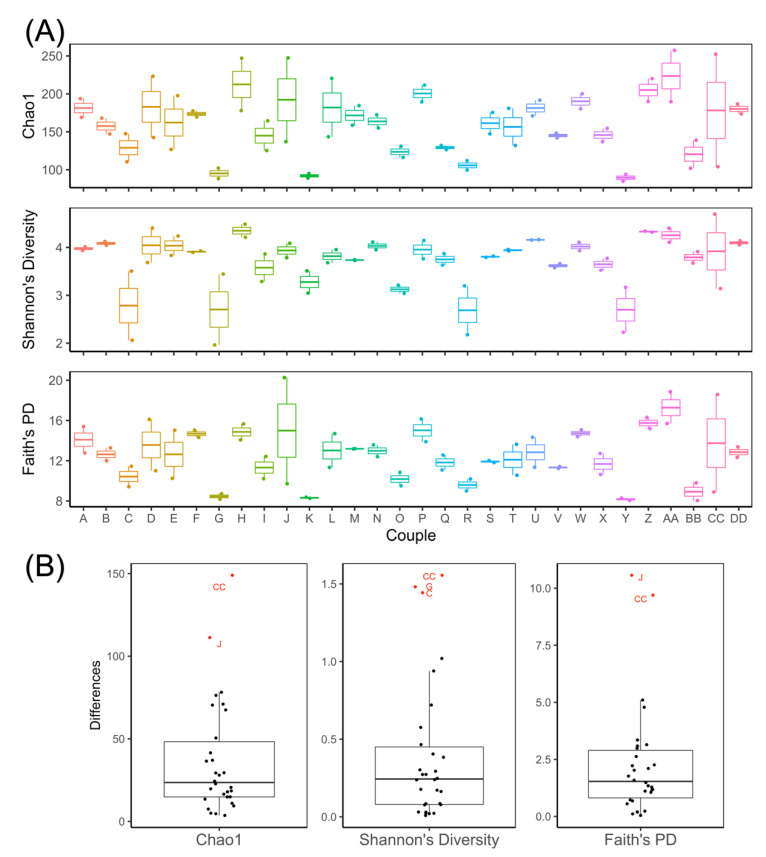
(**A**) Alpha diversity metrics. The labels on the *x*-axis represent anonymized couple identifiers. (**B**) Within-couple differences in alpha diversity metrics. Each point represents the absolute difference of the alpha diversity measures between individuals of one couple. Couples with differences larger than the upper whisker of the boxplot (1.5 times the interquartile range above the hinge) are labeled and were treated as outliers. Faith’s PD: Faith’s phylogenetic diversity.

To examine within-couple concordance versus non-couple concordance, we compared similarity within married couples (30 pairs) to similarity within all possible pairs of individuals not connected by marriage (1740 pairs) on measures of alpha and beta diversity. To reiterate, alpha diversity measures the number of unique taxa (richness) and how equally abundant the taxa are (evenness) in an individual sample; beta diversity measures dissimilarity in microbial composition between samples [54]. We used three alpha diversity metrics: (1) Chao1 [63], which estimates sample richness by considering rare taxa missed from undersampling, (2) Shannon’s diversity [64], which calculates richness and evenness using a natural logarithm, and (3) Faith’s phylogenetic diversity (Faith’s PD) [65], which measures sample richness by incorporating the phylogenetic difference between taxa. As displayed in Figure 1, most individuals shared similar alpha diversity with their partner, except for a subset of four couples who had much larger within-couple differences than other couples (those labeled couples C, G, J, and CC in Figure 1A).

**Figure 2:**
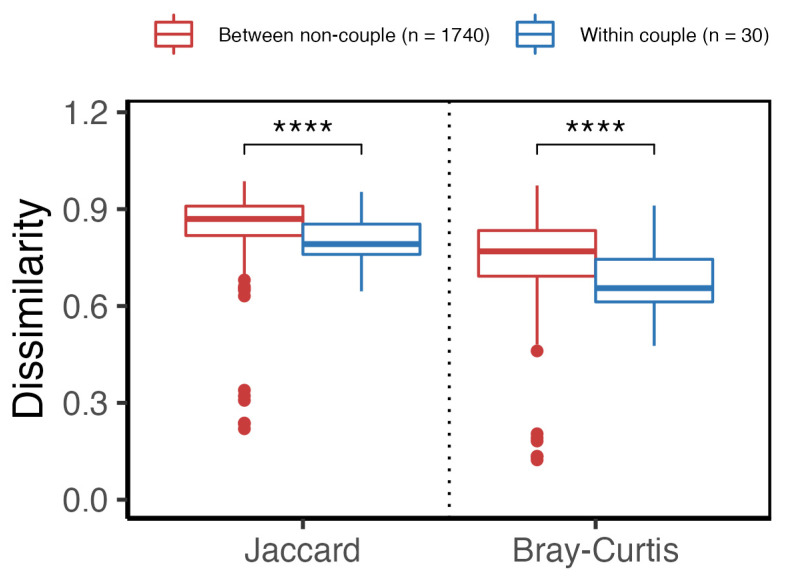
Dissimilarity between each pair of individuals. The “Between non-couple” dissimilarity values were calculated from pairs of non-coupled individuals, and the “Within couple” dissimilarity values were calculated from pairs of couples. The *p*-value above each bracket shows the significance between “Within couple” and “Between non-couple” for each dissimilarity index. ****: *p* < 0.0001. See Supplemental Table S3 for exact *p*-values.

Two common indices were used to assess beta diversity: Jaccard dissimilarity [66] (fraction of distinct taxa between samples, regardless of taxa abundance) and Bray–Curtis dissimilarity [67] (fraction of distinct taxa between samples based on taxa abundance). We calculated dissimilarity between each pair of samples and categorized them into “within-couple” and “between-non-couple”. Jaccard and Bray–Curtis indices showed that within-couple similarity was greater than non-couple similarity (*p* < 0.0001, Figure 2). A downstream cluster analysis using the unweighted pair group method with arithmetic mean (UPGMA) algorithm and Bray–Curtis dissimilarity (Figure S1) further revealed which individuals were closely clustered with their partners and contributed to the significantly smaller dissimilarity. For example, couples B, C, H, K, and DD had the highest within-couple similarity as they fell at the lowest level of the clades. Couples E, F, O, U, X, and Z had relatively low within-couple similarity since they joined at the upper levels of the clades.

**Figure 3:**
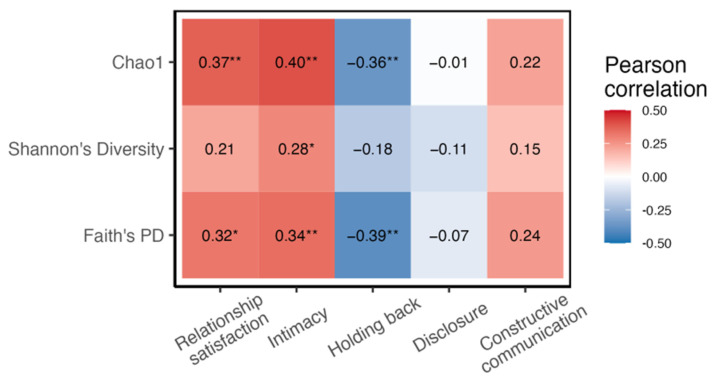
Pearson correlation between alpha diversity metrics and individual psychosocial measures. The value in each color block represents the Pearson correlation coefficient between one alpha diversity metric and one psychosocial measure. ** *p* < 0.01; * *p* < 0.05; Faith’s PD: Faith’s phylogenetic diversity. See Supplemental Table S3 for exact *p*-values.

We next explored associations between the self-reported psychosocial measures listed in Table 2 and gut microbial diversity. We examined correlations between individual measures and each microbial diversity metric. As seen in Figure 3, we observed positive associations of relationship satisfaction and intimacy with the Chao1 and Faith’s PD diversity indices (*p*s < 0.05). Avoidant communication (holding back from disclosure) was inversely related to Chao1 (*p* = 0.0050) and Faith’s PD (*p* = 0.0021). Intimacy was positively associated with Shannon’s diversity (*p* = 0.030); no other relationship measure was related to this metric. Other measures of relationship functioning (disclosure and constructive communication) were not associated with alpha diversity (*p*s > 0.05).

**Figure 4:**
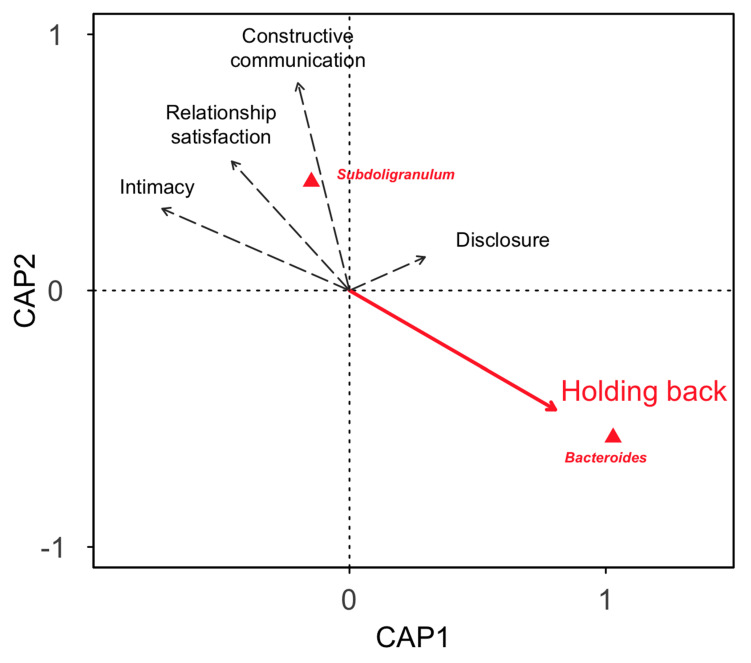
Distance-based redundancy analysis (Bray–Curtis) showing the impact of psychosocial characteristics on the gut microbial composition in older adult couples. The red solid vector represents the psychosocial characteristic (i.e., holding back) that remained after “ordiR2step” variable selection, and the black dashed vectors represent other non-selected psychosocial characteristics. The red triangles and italic labels represent amplicon sequence variants (ASVs) potentially associated with holding back. The other demographic (income, gender) and disease (supplement use) variables were set as conditional variables.

Beta diversity analyses showed that psychosocial characteristics were associated with overall microbial composition (Table S2). Among these characteristics, holding back was most strongly associated with microbial composition variation, as assessed by the Jaccard and Bray–Curtis dissimilarity indices (*p*s < 0.05). We then examined these associations adjusting for potential background confounders. Our relatively small sample size precluded consideration of all potential confounders (demographics, chronic disease, medication, supplement use, and dietary intake); accordingly, we applied a forward variable selection method [68] to identify those most strongly related to dissimilarity (Table S1). Gender identity (female versus male), income (<USD 100k versus >USD 100k), and prebiotic/probiotic supplement use (none versus either or both), but no dietary intake variables (sugar, fiber, or calcium) were selected based on this analysis. We then modeled beta diversity as a function of the relationship characteristics, using the distance-based redundancy analysis (Bray–Curtis). From these measures, only holding back was identified as being related to the microbial composition variation via forward variable selection. This characteristic, as revealed by our linear regression model, was associated with the abundance of amplicon sequence variants (ASVs) belonging to the genera *Bacteroides* and *Subdoligranulum* (unadjusted *p*s = 0.025 and 0.020, respectively; BH-adjusted *p* = 0.044, Figure 4).

## 4. Discussion

To our knowledge, this is the first study of older adult couples designed to examine associations between multiple indicators of relationship functioning and gut microbial composition. Remote recruitment and data collection necessitated by SARS-CoV-2 mitigation efforts proved quite feasible, with all 60 participants successfully completing an online survey and supplying a fecal sample. This suggests that this emerging area of research within the broader arena of social determinants of health is amenable to further scientific examination.

In addition to establishing feasibility, we replicated work showing within-couple concordance in gut microbial composition. Specifically, participants’ gut microbial compositions were found to be more similar to that of their partners than to those of others. This pattern of similarity could be due to microbial sharing through common living spaces/cohabitation, similar diet, and environment, as seen in prior work in both animal and human samples, suggesting that close proximity and social interactions are associated with more similar gut microbial communities [21,22,23,25,29,69]. Recognizing the potential issues posed by unequal sample sizes (*n* = 30 for “within couple” and *n* = 1740 for “between non-couples”), we applied the Brunner–Munzel test to mitigate the potential impact of unequal variances on the test of this difference [60]. Additional research with a larger sample is needed to elucidate the correlates and consequences of within-couple concordance. Despite similarities within couples, we also observed substantial between-individual variation. For instance, one participant from the couple CC had a completely different microbiota compared with other individuals (Figure S1), likely due to a high relative abundance (30%) of the phylum Actinobacteriota (consisting of 66% *Collinsella*, 32% *Bifidobacterium*, and 2% other genera) which was not observed in other individuals (Figures S2 and S3). Three individuals (from couples G, R, and Y) formed a separate cluster (Figure S1), suggesting that they shared similar gut microbiota (e.g., over 50% of *Prevotella*, Figure S3) that were distinct from others.

Our most novel findings are those suggesting associations between gold standard measures of relationship functioning and gut microbial composition. The findings were in predicted directions, such that greater relationship satisfaction and intimacy were associated with greater gut microbial alpha diversity, as were lower levels of holding back. Alpha diversity has been associated with a healthier gut microbiota and a lower risk of chronic disease [17,20,70]. Thus, better relationship functioning could be associated with better gut microbiota and health. Additional research is needed to elucidate mechanisms by which relationship functioning is associated with gut microbial health (e.g., more time spent together and/or more physical contact). Moreover, the finding that holding back from sharing thoughts and feelings was associated with lower alpha diversity (Figure 3) and significant shifts in microbial composition is intriguing. Specifically, holding back was positively associated with the relative abundance of *Bacteroides* ASV and negatively with *Subdoligranulum* ASV (Figure 4). Although *Bacteroides* species play a key role in nutrient metabolism in the human gut, some of them are also recognized as opportunistic pathogens related to conditions such as hypertension and intestinal disorders [71,72]. The abundance of *Subdoligranulum* has been positively associated with a healthy metabolic status [73,74].

A growing body of evidence suggests that holding back from sharing thoughts and feelings with one’s partner is deleterious and associated with greater psychological and relational distress [75,76,77]. Holding back may involve expressive suppression, the act of experiencing a given emotion but suppressing its outward expression [78], e.g., putting on a happy face. Experimental work has demonstrated that expressive suppression is associated with physiological indicators of stress such as increased blood pressure [79]. Holding back could be linked to poorer gut health through repeated activation of the stress response [80], but this is speculative.

Scientific examination of links between social connections and gut microbiota requires an inter-disciplinary approach, drawing on expertise from fields such as psychology, microbiology, gerontology, internal medicine, and gastroenterology, as reflected in this project’s research team, and using established social science methods to gather rich data on couple functioning [81], along with established microbiota assessment and analytic methods to characterize gut microbial composition. Longitudinal designs are warranted to prospectively examine associations among trajectories of communicative exchanges, gut microbial composition, and health outcomes. These would allow for inferences regarding temporal sequencing and lags, and possible identification of bacterial biomarkers.

Conclusions and recommendations put forth by an expert panel in 2019 are highly relevant and critical to consider moving forward [82]. The panel, convened to review evidence regarding “what constitutes a healthy human gut microbiome”, noted large between-person differences in core microbiota structure, as well as large between-person differences in response to “perturbations”, such as dietary changes or the use of probiotics. Accordingly, investigations designed to examine the effect of exposures or interventions should assess the microbiome repeatedly over time and be adequately powered to account for such variability. The panel also determined that clear linkages between changes in gut microbiome structures and health have yet to be elucidated; certainly, that causality has not been established [82].

Because gut microbiota diversity typically declines with age [83] and because close relationships play a critical role in health or lack thereof [3], it is important to study and to develop, test, and provide low-cost effective supportive care for the aging population, to facilitate healthy aging. Social exchanges may modify gut microbiota. Work by Kiecolt-Glaser and colleagues [32] provides a theoretical framework for this emerging area of research by positing pathways linking relationship factors (e.g., conflictual dynamics, life stressors, unhealthy behaviors, and shared spaces) with accelerated aging through changes in gut microbial communities (e.g., low diversity) and resulting physiological sequelae ranging from a leaky gut to inflammation. Pathways in this model are ripe for testing with middle-aged and older adult couples. The findings will inform next-step novel mechanistic studies which could, in turn, inform testing of couple-based communication interventions to improve relational and physical well-being among older adults. Alternatively, interventions could target microbial composition or dietary behavior among those most at risk for health decrements based on relationship quality or other factors. Concordance between partners’ gut microbiota, as well as between their health behaviors, can be capitalized upon to affect change (better communication, improved health behaviors) in not just one but *both* partners and to bolster and leverage dyadic strength and partner support to do so. While increasing in number, few large-scale dyadic interventions have been tested, and none to our knowledge have been tied to gut health.

Our pilot study, designed primarily to test the feasibility of methods and explore patterns of associations, did not include features of a full-scale study. The small sample size, selected to be sufficient for the primary study aims, did not allow for formal hypothesis testing. Moreover, the lack of demographic diversity (92% White, 78% college-educated, 93% different-sex couples) did not allow for meaningful examination of potential moderation of associations by background variables and limits the generalizability of the findings. Further, the cross-sectional design precludes inferences regarding temporal or causal relationships between the psychosocial variables and microbial composition. Finally, we did not assess relationship length or length of cohabitation, which may play important roles in the system of associations linking relationship functioning and microbial diversity [27].

## 5. Conclusions

The results from this pilot study indicate that remote collection of fecal samples from older adult couples is entirely feasible using a kit that allows samples to sit at room temperature for an extended period (see description in the Methods section), that microbial composition within couples is concordant (extending previous research), and that better relationship quality is associated with a healthier microbial composition. These findings contribute to scientific work in this growing area and provide support for current keen interest (at multiple ecological levels and across sectors) [84] in the social aspects of health. More research is needed with human samples, employing rigorous social science methods to identify the extent to which social factors affect gut health in particular, especially in older adults at risk for multiple health decrements, and to tease out pathways linking characteristics of relationships and persons in them to health outcomes. This in turn can inform public health efforts to bolster social connections in this current zeitgeist of challenges including social isolation and loneliness [85].

## Figures and Tables

**Table 1:** Demographic characteristics of the sample.

N	60
Age, M (SD)	66.57 (4.78)
Age, range	60–79
Gender, n (%)	
Male	28 (46.7)
Female	32 (53.3)
Ethnicity, n (%)	
Hispanic or Latinx	1 (1.7)
Not Hispanic or Latinx	58 (96.7)
Unknown	1 (1.7)
Race, n (%)	
Asian	4 (6.7)
White	55 (91.7)
More than one race	1 (1.7)
Educational status, n (%)	
≤High school or GED	2 (3.4)
Some college or technical school	11 (18.3)
4-year college degree	18 (30.0)
Post-baccalaureate degree	29 (48.3)
Income, n (%)	
USD 20k–USD 39,999	2 (3.3)
USD 40k–USD 59,999	10 (16.7)
USD 60k–USD 79,999	16 (26.7)
USD 80k–USD 99,999	2 (3.3)
USD 100k–USD 120,999	10 (16.7)
USD 121k+	20 (33.3)

**Table 2:** Psychosocial and behavioral characteristics of the sample.

Measure	Total
N	60
DAS relationship satisfaction (0–50 scale), M (SD)	39.50 (4.09)
Miller Social Intimacy Scale (1–10 scale), M (SD)	8.13 (1.15)
Emotional Disclosure Scale: disclosure (1–5 scale), M (SD)	2.35 (0.86)
Emotional Disclosure Scale: holding back (1–5 scale), M (SD)	1.66 (0.73)
Communication Patterns Questionnaire: constructive communication (1–9 scale), M (SD)	6.54 (1.39)
Sexual satisfaction in past month (0–10 scale), M (SD)	5.86 (3.65)
Comorbidities, n (%) chronic conditions	
None	34 (56.7)
One	20 (33.3)
Two	5 (8.3)
Three	1 (1.7)
Current medication use, n (%)	
Prilosec	8 (13.3)
Nexium	2 (3.3)
Dexilant	1 (1.7)
Pepcid	5 (8.3)
Current supplement use, n (%)	
Prebiotic	2 (3.3)
Probiotic	9 (15.0)
Dietary intake per Dietary Screener Questionnaire, M (SD)	
Fiber, g/day	16.97 (2.61)
Calcium, mg/day	985.80 (202.33)
Added sugars, teaspoon equivalents/day	15.62 (6.15)

## Data Availability

The raw sequence data were deposited in the National Center for Biotechnology Information Sequence Read Archive database (BioProject accession #PRJNA928837). De-identified questionnaire data are available upon request to the corresponding author.

## Supplementary Materials

The following supporting information can be downloaded at https://www.mdpi.com/article/10.3390/ijerph20085435/s1. Table S1: Variable selection; Table S2: Permutational analysis of variance showing associations between two beta diversity indices and the psychosocial measures; Table S3: *p*-value summary for Figure 2 and Figure 3; Figure S1: Unweighted pair group method with arithmetic mean (UPGMA) dendrogram showing the relationships of gut microbiota composition among the 60 participants based on the Bray–Curtis dissimilarity index; Figure S2: Relative abundance of gut microorganisms at the phylum level; Figure S3: Relative abundance of gut microorganisms at the genus level.
