# Supplementary material for: Relationship Functioning and Gut Microbiota Composition among Older Adult Couples
Source: Int J Environ Res Public Health. 2023 Apr 7;20(8):5435. doi: 10.3390/ijerph20085435 (PMC10138905; doi:10.3390/ijerph20085435)

Figure S2. Relative abundance of gut microorganisms at the phylum level. Letters on top of the panels represent couple IDs, and labels on the x axis represent the two participants within each couple.

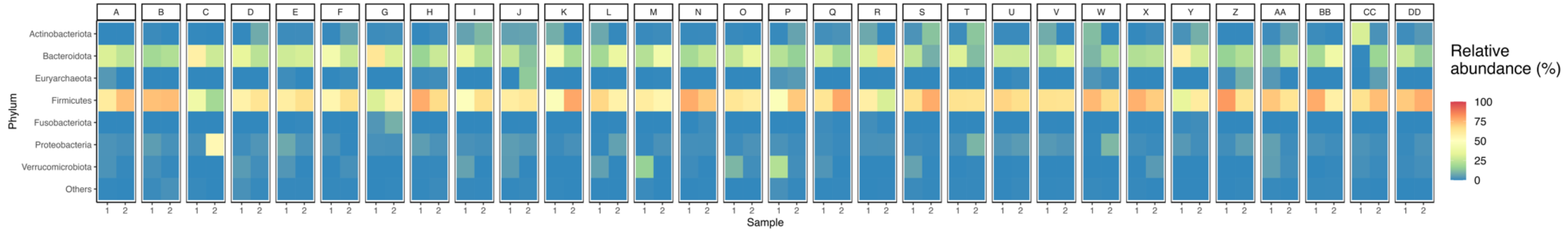

Supplement: Supplementary file 1 [file ijerph-20-05435-s001.zip › Figure S2.pdf]
