# Supplementary material for: Relationship Functioning and Gut Microbiota Composition among Older Adult Couples
Source: Int J Environ Res Public Health. 2023 Apr 7;20(8):5435. doi: 10.3390/ijerph20085435 (PMC10138905; doi:10.3390/ijerph20085435)
Supplement: Supplementary file 1 [file ijerph-20-05435-s001.zip › Table S1.pdf]

**Table S1**. Variable selection.

| Categories of measures | Measures included in 1st "capscale" | Measures selected in 1st "ordiR2step" | Measures included in 2nd "capscale" | Measures selected in 2nd "ordiR2step" |
|---|---|---|---|---|
| Psychosocial | • Relationship satisfaction<br>• Intimacy<br>• Holding back<br>• Disclosure<br>• Constructive communication | • Holding back | • Relationship satisfaction<br>• Intimacy<br>• Holding back<br>• Disclosure<br>• Constructive communication | • Holding back |
| Demographic | • Age<br>• Gender<br>• Education<br>• Income | • Gender<br>• Income | • Gender<br>• Income<br>(set as "Condition") | |
| Sexual satisfaction | • Sexual satisfaction | | | |
| Medication and chronic disease | • Proton pump inhibitor use<br>• Supplement use<br>• Chronic disease | • Supplement use | • Supplement use<br>(set as "Condition") | |
| Dietary intake | • Added sugar<br>• Calcium<br>• Fiber | | | |
