# Supplementary material for: Relationship Functioning and Gut Microbiota Composition among Older Adult Couples
Source: Int J Environ Res Public Health. 2023 Apr 7;20(8):5435. doi: 10.3390/ijerph20085435 (PMC10138905; doi:10.3390/ijerph20085435)
Supplement: Supplementary file 1 [file ijerph-20-05435-s001.zip › Table S2_revised.pdf]

Table S2. Permutational analysis of variance showing associations between two beta diversity ~~distances~~ indices and the psychosocial measures. P values less than 0.05 are bolded.

Measure	~~Distance~~Beta diversity index	Adonis2 R^2	Adonis2 significance
Relationship satisfaction	Jaccard	2.1e-02	5.5e-02
	Bray-Curtis	2.3e-02	6.3e-02
Intimacy	Jaccard	2.0e-02	1.2e-01
	Bray-Curtis	2.2e-02	1.0e-01
Holding back	Jaccard	2.3e-02	**2.2e-02**
	Bray-Curtis	2.6e-02	**2.3e-02**
Disclosure	Jaccard	1.6e-02	6.6e-01
	Bray-Curtis	1.5e-02	7.0e-01
Constructive communication	Jaccard	2.0e-02	8.3e-02
	Bray-Curtis	2.3e-02	6.3e-02
