# Supplementary material for: Relationship Functioning and Gut Microbiota Composition among Older Adult Couples
Source: Int J Environ Res Public Health. 2023 Apr 7;20(8):5435. doi: 10.3390/ijerph20085435 (PMC10138905; doi:10.3390/ijerph20085435)
Supplement: Supplementary file 1 [file ijerph-20-05435-s001.zip › Table S3.pdf]

Table S3. *P* value summary for Figures 2 and 3.

Figure	Comparisons/correlations	*P* value
2	Between non-couple vs Within couple:	
	Jaccard	0.000017
	Bray-Curtis	0.000017
3	Chao1	
	Relationship satisfaction	0.0039
	Intimacy	0.0017
	Holding back	0.0050
	Disclosure	0.95
	Constructive communication	0.094
	Shannon’s Diversity	
	Relationship satisfaction	0.11
	Intimacy	0.030
	Holding back	0.16
	Disclosure	0.41
	Constructive communication	0.26
	Faith’s PD	
	Relationship satisfaction	0.012
	Intimacy	0.0087
	Holding back	0.0021
	Disclosure	0.62
	Constructive communication	0.064
