# Supplementary material for: Relationship Functioning and Gut Microbiota Composition among Older Adult Couples
Source: Int J Environ Res Public Health. 2023 Apr 7;20(8):5435. doi: 10.3390/ijerph20085435 (PMC10138905; doi:10.3390/ijerph20085435)

Figure S1. Unweighted pair group method with arithmetic mean (UPGMA) dendrogram showing the relationships of gut microbiota composition ~~similarity~~ among the 60 participants based on the Bray-Curtis ~~distance~~dissimilarity index. Clade labels represent couple IDs. The IDs in red represent couples with the highest within-couple similarity (i.e., joining at the lowest level of the clades), and the ones in blue represent those with relatively smaller similarity (joining at upper levels of the clades). The IDs in black represent couples with no similarity.

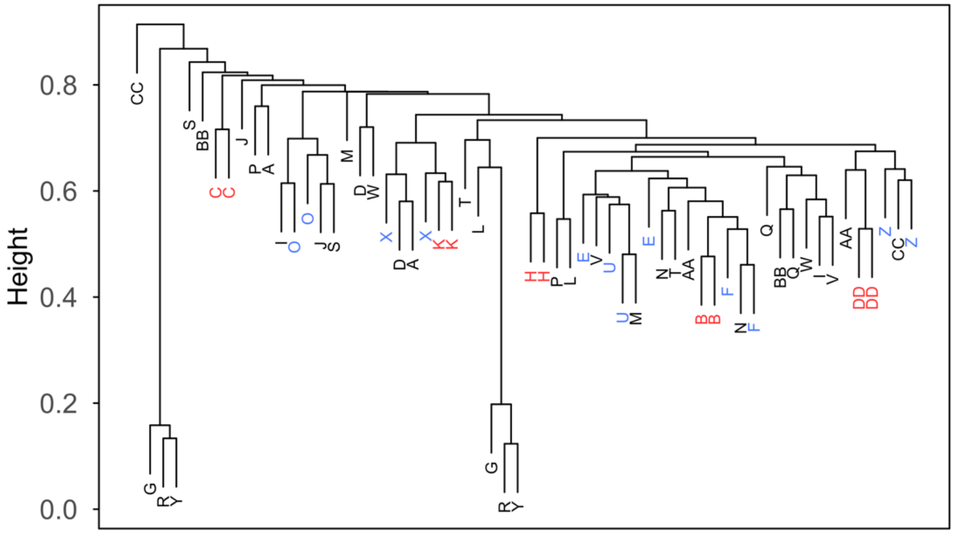

Supplement: Supplementary file 1 [file Figure S1_revised.pdf › Figure S1_revised.pdf]
